# Starting the Transition Towards Integrated Community Care 4all

**DOI:** 10.5334/ijic.5553

**Published:** 2020-06-30

**Authors:** Tinne Vandensande

**Affiliations:** 1 King Baudouin Foundation, a public benefit foundation, on behalf of the TransForm Partnership, BE

**Keywords:** Local Communities, Social Capital, Resilience, Place-based Governance, Primary Care, Integrated Care, Transform

Integrated Community Care (ICC) is a new concept that has been launched by the international partnership of philanthropic organisations known as TransForm which came into being in 2018. The TransForm partners are convinced of the value of investing time, resources and imagination to enhance the capacity of local communities to deal with public health issues and the care needs of community members throughout their whole lifetime.

The TransForm initiative is conceived as a learning journey, since this is the only way to move forward while maintaining a longitudinal perspective and creating space for the necessary learning curve. Our hope is that sharing our reasoning and insights will allow interested parties to understand how we can support ICC and embed and express it in practice, find the storylines that form the ‘ICC 4all’ narrative and identify principles that could help in building sustainable and context-sensitive ICC.

ICC has an undeniable relevance to all stakeholders and a high face validity. It brings together three generic concepts: “integrated”, “community” and “care”. In its most rudimentary form, ICC is recognized as a much-needed and valuable expansion of more widespread notions of integrated care, explicitly recognising the value, potential and power of communities, citizens and laypeople. Going even deeper than this, however, ICC deserves its own approach. Strengthening communities will require a fundamental shift in the way we value and understand the role of people and communities as an integral part of the system. It involves a shift from an ego-system awareness with the emphasis on one’s own well-being, to an eco-system awareness that simultaneously emphasises the well-being of all [5].

Integrated community care is now more firmly on the agenda than ever. Citizens, neighbourhood networks, community-based organizations and informal carers are all being recognized as key players in tackling the Covid-19 crisis as they address the huge needs for psychosocial, practical and food support [1]. Bottom-up initiatives are popping up to respond to the needs of the most vulnerable individuals in our communities. The response to the crisis from the voluntary sector has been spectacular. Their example inspires us to act differently, cooperate better and innovate faster. Cooperation and innovation are necessary if we are to increase the resilience of our health and care systems [2].

ICC acknowledges that communities are essential partners that contribute invaluable assets: relationships, expertise, contextual knowledge, entrepreneurship, public space and services, and locally supportive ecosystems. ICC seeks to create conditions that will allow people to care for themselves and also for others in their community. It is a whole-of-society approach to health and well-being, centred on the needs and preferences of citizens, families and communities. One key feature of ICC is that it moves away from ‘delivery’ and towards genuine ‘co-development’. Individuals and communities are no longer framed as recipients. This gives it a greater relevance as it aligns with fundamental shifts taking place in society and in health and care systems. ICC is linked to a positive, empowering understanding of health. It encompasses the desire to be a positive force for change amid the multi-faceted transition to a new, sustainable equilibrium for our societies. The main elements of the narrative can be summarised as follows:

✓ ICC engages and empowers people in local communities;
✓ ICC promotes a sense of accountability towards a territorially defined population;
✓ ICC fosters inclusiveness and reaching out to underserved and marginalised groups;
✓ ICC activates and reinforces the social ties between people;
✓ ICC is goal-oriented in nature, supporting people’s priorities and life goals;
✓ ICC strengthens communities by tackling social, economic and environmental determinants of health;
✓ ICC comes down to a continuous process of whole-system innovation;
✓ ICC requires a social movement to make it a reality.

There is no single model for operationalizing ICC but a wide range of existing practices express the DNA inherent within the concept in different ways. These include, among others, Community Health Centres, Vibrant Communities and Healthy Place-making [6].

TransForm, the international coalition for learning in the area of ICC, has identified three storylines to guide us as we seek ways to make ICC the new standard of care:

- **A systems storyline** to fully acknowledge the heterogeneity of ICC and to map and understand the many drivers and strategies behind the various models and practices that exist in integrated community care.
- **A participatory storyline** to emphasize the importance of a shared vision, a strong narrative and the active involvement of all at the micro, meso and macro levels in communities and in society.
- **An implementation storyline** to focus on the formulation of meaningful and effective guiding principles that can help ICC to mature and become more robust in diverse contexts, amidst changing views and evolving challenges. The principles are intended to provide direction to those aiming to make ICC a reality.

The principles underpinning effectiveness adhere to the GUIDE criteria: they help with Guidance (priority setting), they have Utility (i.e. they are actionable), they are Inspiring (motivating people to ‘walk the talk’), they are Developmental (i.e. applicable to a range of contexts) and they are Evaluable (i.e. you can document and judge the results).

The seven effectiveness principles for Integrated Community Care are set out below. These are grouped into three categories: the importance of authentic partnerships, the relevance of facilitating and enabling infrastructure and the dynamics of evaluation [3]:

## Co-develop health and wellbeing, enable participation

1. Value and foster the capacities of all actors in the community, including citizens, to become change agents and co-produce health and wellbeing. This requires the active involvement of all actors, paying special attention to the most vulnerable ones.
2. Foster the creation of local alliances among all actors involved in the production of health and wellbeing in the community. Develop a shared vision and common goals. Actively work towards balanced power relations and mutual trust within these alliances.
3. Strengthen community-oriented primary care that enhance people’s capabilities to maintain health and/or to live in the community with complex chronic conditions. Use people’s life goals as the starting-point to define the desired outcomes of care and support.

## Build resilient communities

4. Improve the health of the population and reduce health disparities by addressing the social, economic and environmental determinants of health in the community and investing in prevention and health promotion.
5. Support healthy and inclusive communities by providing opportunities to bring people together and by investing in both social care and social infrastructure.
6. Develop the legal and financial conditions to enable the co-creation of care and support at the community level.

## Monitor, evaluate and adapt

7. Continuously evaluate the quality of care and support and the status of health and wellbeing in the community, using methods and indicators based on which are grounded within the foregoing principles and documented using a ‘community diagnosis’ involving all stakeholders, with wide participation. Provide opportunities for joint learning. Adapt policies, services and activities in accordance with the evaluation outcomes.

The Covid-19 crisis may be a turning-point. It is giving us a unique opportunity to acknowledge ICC as a systemic approach that blurs boundaries between informal and formal care, between different skills in the workforce and between primary and specialist care. We call on all stakeholders and policymakers to work together to find inclusive and sustainable solutions that can move us towards ‘ICC 4all’:

¬ By creating and supporting an international (research) community of changemakers, maintaining the impetus towards ICC and driving progress.
¬ By avoiding competition between existing models, initiatives or ‘brands’, instead supporting existing knowledge, local movements and advocates.
¬ By providing structural funding so that countries and regions are supported through a transition phase.
¬ By facilitating place-based governance and accountability to a territorially defined population.
¬ By studying the practical aspects and opportunities involved in establishing local and neighbourhood trusts, so that public and private financing can be pooled at the local level.
¬ By establishing the role of primary care as the preferred point of access, gatekeeper and safety net for the community.
¬ By recognizing that ICC is a societal process, not just a professional or managerial toolbox.

The ongoing Covid-19 pandemic has revealed the fragilities in key infrastructures and systems. A renewed emphasis on resilience creates opportunities for a more richly textured and locally sensitive health and social care system that plays to the strengths of citizens and communities and addresses social and environmental determinants of health [4].

The TransForm experience has already generated insights into a new paradigm that may bring fundamental benefits for all people, especially for under-served and marginalised groups. Nevertheless, we must have patience with the slow pace of fast change. We believe in the power of experimentation, in working continuously to build our evidence base and in ongoing enlargement of our learning coalition.

## Author Information

This editorial is written on behalf of the TransForm Partnership, an international forum intended to foster integrated community care in Europe and beyond: https://transform-integratedcommunitycare.com.
